# Adaptive Capacity of a DNA Polymerase Clamp-loader ATPase Complex

**DOI:** 10.1093/molbev/msae013

**Published:** 2024-01-31

**Authors:** Subu Subramanian, Weilin Zhang, Siddharth Nimkar, Mazzin Kamel, Michael O’Donnell, John Kuriyan

**Affiliations:** Department of Biochemistry, School of Medicine, Vanderbilt University, Nashville, TN, USA; Department of Chemistry, University of California, Berkeley, Berkeley, CA, USA; Department of Biochemistry, School of Medicine, Vanderbilt University, Nashville, TN, USA; Department of Molecular and Cell Biology, University of California, Berkeley, Berkeley, CA, USA; Howard Hughes Medical Institute, The Rockefeller University, New York, NY, USA; Department of Biochemistry, School of Medicine, Vanderbilt University, Nashville, TN, USA; Department of Chemistry, Vanderbilt University, Nashville, TN, USA

**Keywords:** phage evolution, conditional neutrality, clamp loader, protein evolution

## Abstract

The ability of mutations to facilitate adaptation is central to evolution. To understand how mutations can lead to functional adaptation in a complex molecular machine, we created a defective version of the T4 clamp-loader complex, which is essential for DNA replication. This variant, which is ∼5,000-fold less active than the wild type, was made by replacing the catalytic domains with those from another phage. A directed-evolution experiment revealed that multiple substitutions to a single negatively charged residue in the chimeric clamp loader—Asp 86—restore fitness to within ∼20-fold of wild type. These mutations remove an adventitious electrostatic repulsive interaction between Asp 86 and the sliding clamp. Thus, the fitness decrease of the chimeric clamp loader is caused by a reduction in affinity between the clamp loader and the clamp. Deep mutagenesis shows that the reduced fitness of the chimeric clamp loader is also compensated for by lysine and arginine substitutions of several DNA-proximal residues in the clamp loader or the sliding clamp. Our results demonstrate that there is a latent capacity for increasing the affinity of the clamp loader for DNA and the sliding clamp, such that even single-point mutations can readily compensate for the loss of function due to suboptimal interactions elsewhere.

## Introduction

The evolutionary divergence of proteins is dependent on the ability of mutations to modulate protein function to better align with changing conditions. Protein machines have a remarkable capacity to accept mutations without complete loss of function and to optimize fitness under changing external constraints. For machines that are critical to the most central processes of life, an important question is how this adaptation is accomplished without extinguishing the capacity of the system to continue living. In this study, we investigate the adaptive capacity of one such critically important protein machine, the DNA polymerase clamp-loader complex.

**Fig. 1.**
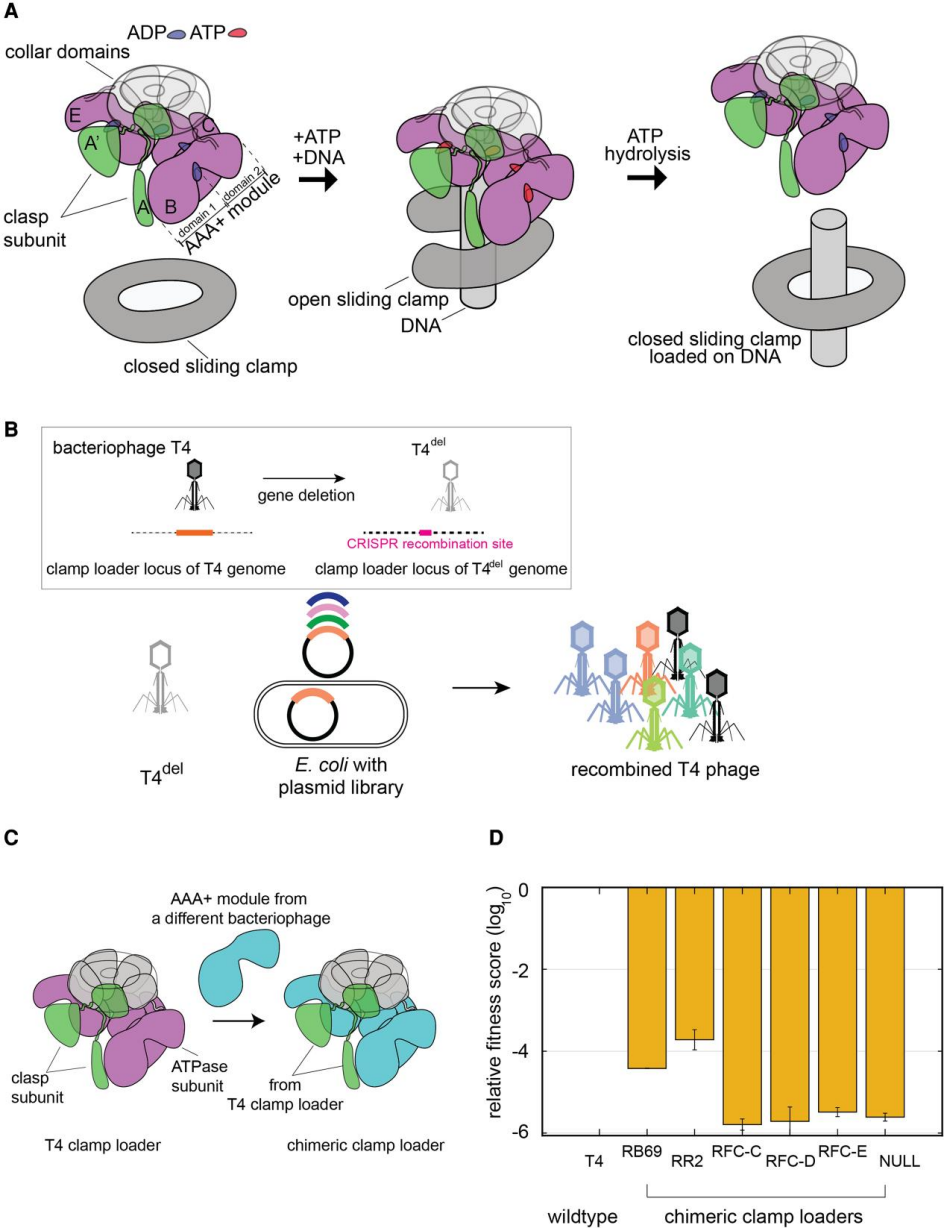
A phage-propagation assay to measure the fitness of clamp-loader variants in a T4 bacteriophage. a) A schematic diagram of the clamp-loading cycle, from left to right, showing key steps of loading the sliding clamp around a primer-templated DNA. b) A schematic of the phage-propagation assay in which T4^del^ infects host cells, each of which carries a plasmid-borne copy of variant genes of the T4 clamp loader. Upon infection, the variant genes recombine into the genome of T4^del^, and this genome is replicated using the proteins encoded by the variant genes. The number of copies of the recombinant genomes (and the recombinant phage) is proportional to the activity of the clamp-loader variant in the host cell. Inset: A method to generate T4^del^ from the wild-type T4 bacteriophage using CRISPR-based genome engineering. c) A schematic diagram showing the construction of chimeric clamp-loader variants. d) The relative fitness of the chimeric clamp-loader variants from the phage-propagation assay.

During genome replication, clamp-loader complexes load ring-shaped sliding clamps onto primer-templated DNA (Kelch et al. 2012). Sliding clamps enable highly processive DNA replication by anchoring the DNA polymerases onto DNA. The clamp-loader cycle, illustrated in Fig. 1a, involves 3 steps: ATP-bound clamp loader binding and opening of the sliding clamp, recognition and binding of the clamp-loader/clamp complex on DNA, and cooperative ATP hydrolysis, leading to the release of the closed clamp around DNA (Kelch 2016).

Our work uses T4 bacteriophage as a model system for understanding the mutational response of the DNA replication machinery. The T4 phage genome encodes a complete set of replication proteins, including a replicative DNA polymerase and a clamp-loader/clamp system. The pentameric T4 clamp-loader complex comprises 4 identical ATPase subunits (gp44, denoted B to E in Fig. 1a), encoded by gene 44, and the clasp subunit (gp62, subunit A in Fig. 1a) encoded by gene 62 (Kelch et al. 2011). Clamp-loader subunits consist of N-terminal AAA+ modules and C-terminal collar domains that are responsible for maintaining the pentameric assembly (Guenther et al. 1997; Jeruzalmi et al. 2001; Bowman et al. 2004; Kelch et al. 2011). ATP binds at interfacial sites between neighboring AAA+ modules. The binding of primer-template DNA in a central channel formed by the AAA+ modules and the sliding clamp triggers the hydrolysis of ATP and the dissociation of the clamp loader from the sliding clamp, resulting in a release of the clamp around DNA (Simonetta et al. 2009; Kelch et al. 2012; Marzahn et al. 2014; Liu et al. 2017; Zheng et al. 2022).

We developed a high-throughput T4 phage-propagation assay to measure the fitness of replication genes in their natural context (Fig. 1b; Subramanian et al. 2021). The phage-propagation assay utilizes a genetically modified T4 phage, referred to as T4^del^, from which the genes encoding the sliding clamp (gene 45) and the clamp-loader subunits (genes 44 and 62) have been deleted (Fig. 1b, inset). Although T4^del^ phage particles can infect *Escherichia coli* cells successfully, the phage genome cannot be replicated because of the missing components in the replication machinery. In the assay, T4^del^ is used to infect an *E. coli* cell library in which each cell carries plasmid-borne variants of the missing replication genes. Upon phage infection, variant genes from the plasmid are copied into the genome of T4^del^ using a CRISPR-Cas12a system. The number of phage particles produced from a bacterial cell is proportional to the function of the variant genes expressed in that cell. By measuring the frequency of each variant gene in the population using Illumina sequencing—in the plasmid library before phage infection and in the recombined phage population after infection—the fitness of each variant can be calculated.

We used the phage-propagation assay to perform a deep mutational scan of the clamp-loader complex and found that the clamp loader exhibits high mutational tolerance across most of its structure (Subramanian et al. 2021). Except for a sparse set of spatially contiguous residues in the functional core of the protein, which are involved in ATP binding and hydrolysis and DNA recognition (constituting ∼10% of the clamp-loader complex), most residues in the clamp-loader complex tolerate point mutations without substantial loss in fitness. The mutational data also showed that the fitness of the wild-type clamp loader is not improved further by point mutations, suggesting that under normal conditions of bacterial growth, phage propagation is not limited by clamp-loader function (Subramanian et al. 2021).

An important question in understanding adaptive processes in molecular evolution concerns the capacity of proteins to adapt to changes in their environment or to recover from mutational damage. We investigate the adaptive capacity of a defective variant of the clamp loader in terms of its ability to rescue the functional defect during phage propagation. The variant clamp loader, which is a chimera created by replacing the AAA+ modules of the T4 clamp loader with the corresponding modules from another clamp loader, is barely able to support phage replication and thus provides a sensitized genetic background that enables us to identify gain-of-function mutations. We subjected the phage bearing the chimeric complex to directed evolution and saturation point mutagenesis and identified mutations that compensate for the decreased fitness of the chimeric clamp-loader variant.

## Results and Discussion

### Engineering a Clamp-loader Variant with a Substantially Reduced Fitness

The structures of the AAA+ modules of clamp loaders are conserved across all branches of life, but their sequences have diverged considerably. The sequence of the AAA+ modules in bacteriophage clamp loaders has pairwise sequence identities as low as 25% (based on the alignment reported in Subramanian et al. (2021)). In eukaryotic clamp loaders (replication factor C [RFC] complex), each subunit of the pentameric assembly is encoded by a different gene. This has allowed the AAA+ modules in each subunit to further specialize in function and diverge in sequence (Schmidt et al. 2001). The pairwise sequence identities between the AAA+ module of the T4 clamp loader and each of the AAA+ modules of the yeast RFC complex range from 19% to 26% (supplementary fig. S1a, Supplementary Material online).

To generate a variety of clamp loaders with different degrees of loss of fitness, we created chimeric clamp loaders in which the AAA+ module of the ATPase subunit of the T4 clamp loader (residues 1 to 230) was replaced by corresponding modules from other clamp loaders (Fig. 1c). We then used the phage-propagation assay to rank-order the chimeric variants by fitness. The chimeric clamp-loader variants generated in this fashion utilize the sliding clamp and other components of the replication system from the T4 phage. Therefore, any reduction in fitness that results from the propagating phage using the chimeric variants is due to nonoptimal interactions between the transplanted AAA+ modules and the rest of the T4 replication machinery or due to inefficiencies intrinsic to the AAA+ module itself.

Our creation of these chimeric clamp loaders is an artificial strategy that decreases fitness by swapping in catalytic domains from a different clamp loader. We note that natural recombination events within phage genomes can potentially yield clamp loaders with chimeric configurations, akin to the variants discussed above. When 2 different phage particles infect a host cell, the genomes of the progeny are mosaics of the parents due to extensive recombination (Hendrix et al. 1999; Hatfull 2008). While this is possible, the work presented here is motivated by an interest in studying how protein systems adapt in response to a reduction in fitness, rather than a specific interest in these chimeric systems.

We tested 5 chimeric clamp-loader variants using the phage-propagation assay. Two variants were generated by using AAA+ modules from bacteriophage clamp loaders: one from RB69 phage (83% sequence identity to the T4 AAA+ module) and one from *Aeromonas salmonicida* phage 44RR2.8t (denoted as RR2 phage [Nolan et al. 2006; Petrov et al. 2006], with 64% sequence identity to the T4 AAA+ module; supplementary fig. S1a, Supplementary Material online). Three chimeric variants were generated with AAA+ modules from a eukaryotic system (the yeast RFC complex), each having <30% sequence identity to the T4 AAA+ module (supplementary fig. S1a, Supplementary Material online).

We used the phage-propagation assay to measure the ability of the chimeric clamp-loader variants to support the replication of the T4 phage (Fig. 1b). We calculate the relative fitness (*F*) of each variant in the assay as follows:


$$\begin{array}{rl} F\;= & \log_{10}\left(\frac{{\mathrm{variant}}^{\mathrm{output}\;\mathrm{counts}}}{{\mathrm{variant}}^{\mathrm{input}\;\mathrm{counts}}}\right)\\ & -\log_{10}\left(\frac{{\mathrm{reference}}^{\mathrm{output}\;\mathrm{counts}}}{{\mathrm{reference}}^{\mathrm{input}\;\mathrm{counts}}}\right) \end{array}$$

where *F* is the relative fitness score for the variant relative to the reference (T4 clamp loader in this experiment). The outputcounts correspond to the number of counts observed in the recombinant-phage library for the variant and reference clamp-loader sequences. The inputcounts correspond to the number of counts observed in the starting plasmid library for the variant and reference. A variant with a relative fitness score of 0 propagates at the same rate as the wild-type clamp loader. Variants with fitness scores of −1 and −2 propagate 10-fold and 100-fold slower, respectively, than the reference.

We used the phage-propagation assay to measure the relative fitness of the chimeric clamp-loader variants (Fig. 1d). The 3 variants with yeast RFC AAA+ modules are essentially nonfunctional, with relative fitness scores that are comparable with that of the null variant (a variant in which the 3 codons following the start codon of gene 44 are replaced by stop codons). Chimeric variants generated using AAA+ modules from RB69 and RR2 phage clamp loaders have fitness scores that are several orders of magnitude lower than that of the wild-type clamp loader but are still measurable (the propagation rates are ∼5 × 10^4^-fold and ∼5 × 10^3^-fold lower than wild type, respectively). In a separate experiment where the wild-type T4 phage was competed with T4^del^ recombined with the RR2-chimeric clamp loader, a similar fitness difference between the 2 variants was observed. This suggests that the influence of the variant clamp loader on the CRISPR-mediated recombination can be disregarded.

To identify mutations in chimeric clamp loaders that result in increased fitness, we focused on the variant with the AAA+ module from the RR2 phage (the RR2/T4 chimera) since it is more divergent in sequence compared with the AAA+ module from the RB69 phage (64% vs. 83% sequence identity). Of the 230 residues in the AAA+ module, 83 are different between the RR2 and the T4 sequences (supplementary fig. S2a, Supplementary Material online). Using previously determined mutational data for the T4 clamp loader (Subramanian et al. 2021), we compared the distribution of fitness effects for each of these 83 substitutions with the T4 clamp-loader sequence, taken individually, with the distribution of all possible single amino acid substitutions in the T4 background (supplementary fig. S2b, Supplementary Material online). The analysis shows that 80 of the 83 substitutions, when made individually in the wild-type context, have a marginal effect (<3-fold) on T4 phage propagation. Three substitutions (H44N, G209N, and S213F) result in a moderate (∼10-fold) reduction in phage propagation. Mutations of Gly 86, which we focus on in the discussion below, have only mild effects (<3-fold decrease in phage propagation) when introduced in the context of the wild-type clamp loader (supplementary fig. S3, Supplementary Material online).

### Directed Evolution to Recover Fitness in the RR2/T4 Chimeric Clamp Loader

**Fig. 2.**
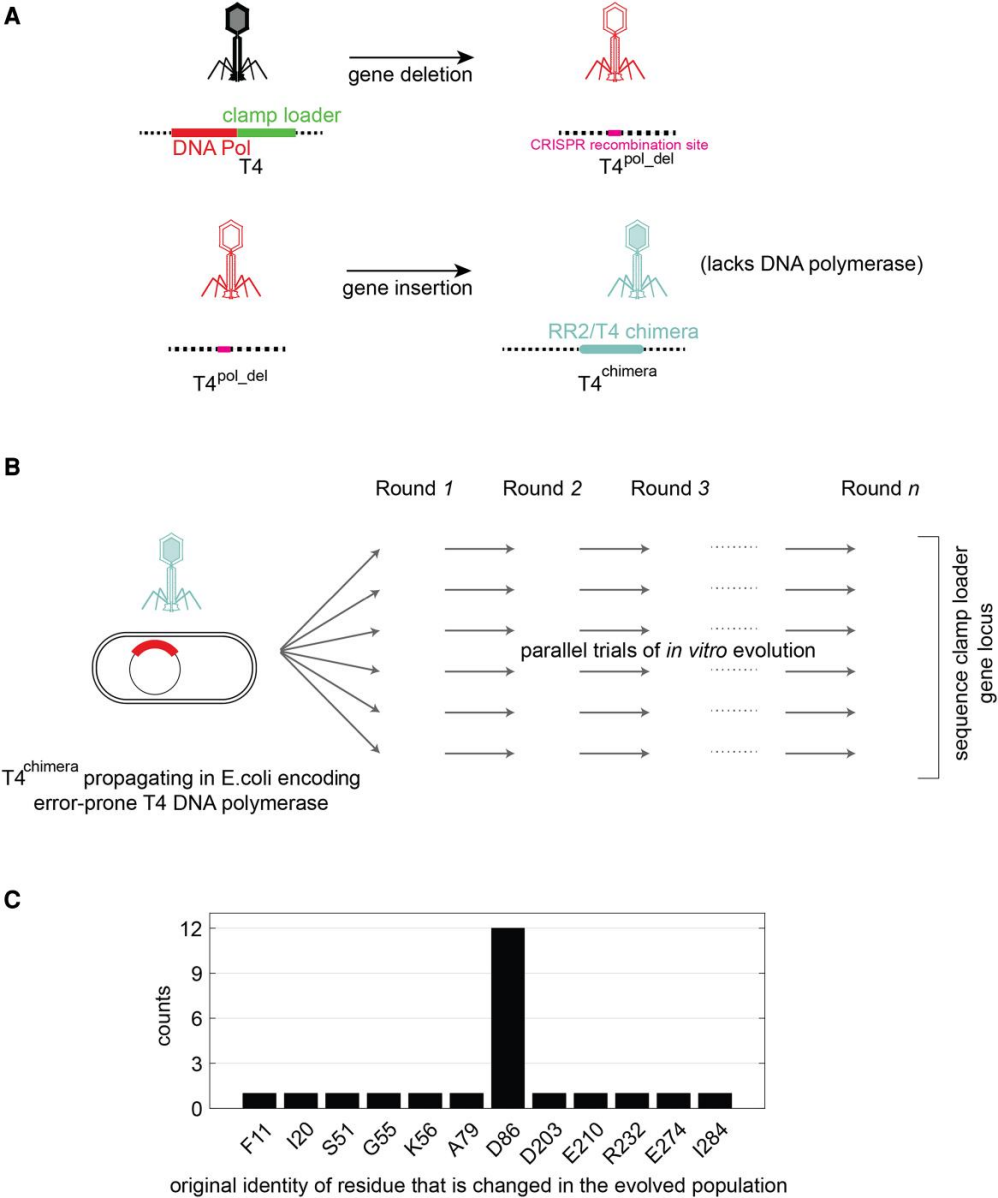
A platform for performing in vitro evolution to improve the fitness of bacteriophage T4 propagation using the RR2/T4 chimeric clamp loader. a) A schematic diagram depicting the construction of the T4^chimera^ used to perform in vitro evolution. b) A schematic representation of the in vitro evolution protocol used in the study. c) Mutations identified by subjecting the T4^chimera^, bearing the RR2/T4 chimeric clamp loader, to in vitro evolution.

We modified the phage-propagation platform to carry out directed-evolution experiments on the RR2/T4 chimeric clamp loader. We used the CRISPR-Cas12a genome editing system (Zetsche et al. 2015) to generate a new strain of T4 phage—referred to as T4^pol_del^ phage—in which the genes encoding the T4 DNA polymerase (gene 43) and the T4 clamp loader (genes 44 and 62) are deleted (Fig. 2a; for details, see Materials and Methods). We then inserted genes encoding the RR2/T4 chimera into the T4^pol_del^ phage to generate the T4^chimera^ phage (Fig. 2a). The T4^chimera^ phage lacks a gene for DNA polymerase and can propagate only in host cells harboring a plasmid-borne copy of the T4 DNA polymerase gene.

When propagated, the T4^chimera^ phage produces new T4^chimera^ phage particles (devoid of the DNA polymerase gene) in numbers that are proportional to the activity of the chimeric clamp-loader variant. In order to perform in vitro evolution, T4^chimera^ phages are propagated over many rounds (10 to 15 rounds in this study), so that individual phage genomes that spontaneously acquire beneficial mutations get enriched in the phage population. The rate of finding beneficial mutations can be accelerated by increasing the overall mutation rate, through the use of error-prone variants of T4 DNA polymerase (Langhorst et al. 2012). We used a single-mutant variant of the T4 DNA polymerase (D219A), with a ∼1,000-fold higher mutation rate than the wild-type polymerase (Frey et al. 1993), to passage the phage through multiple rounds of in vitro evolution.

We carried out 15 independent trials of in vitro evolution with the error-prone variant of T4 DNA polymerase. For each trial, after 10 rounds of passaging the phage, we sequenced the genes encoding the clamp loader and the sliding clamp in the phage population using Sanger sequencing and identified the dominant mutations. A total of 23 point mutations were identified in this way. Strikingly, 12 of the 15 trials identified mutations to a single residue, Asp 86, in the AAA+ module of the RR2/T4 chimera, with all other mutations observed in only one of the trials (Fig. 2b and supplementary fig. S4a, Supplementary Material online).

We observed that substitutions at residue Asp 86 of the AAA+ module of the chimeric clamp loader first appear in the population at different rounds, but once they appear, they immediately take over the population in the subsequent round of phage propagation (representative sequencing data are given in supplementary fig. S4b and c, Supplementary Material online), suggesting that these mutations result in a substantial increase in the fitness of the T4^chimera^.

### The Fitness Effects of Asp 86 Substitutions

**Fig. 3.**
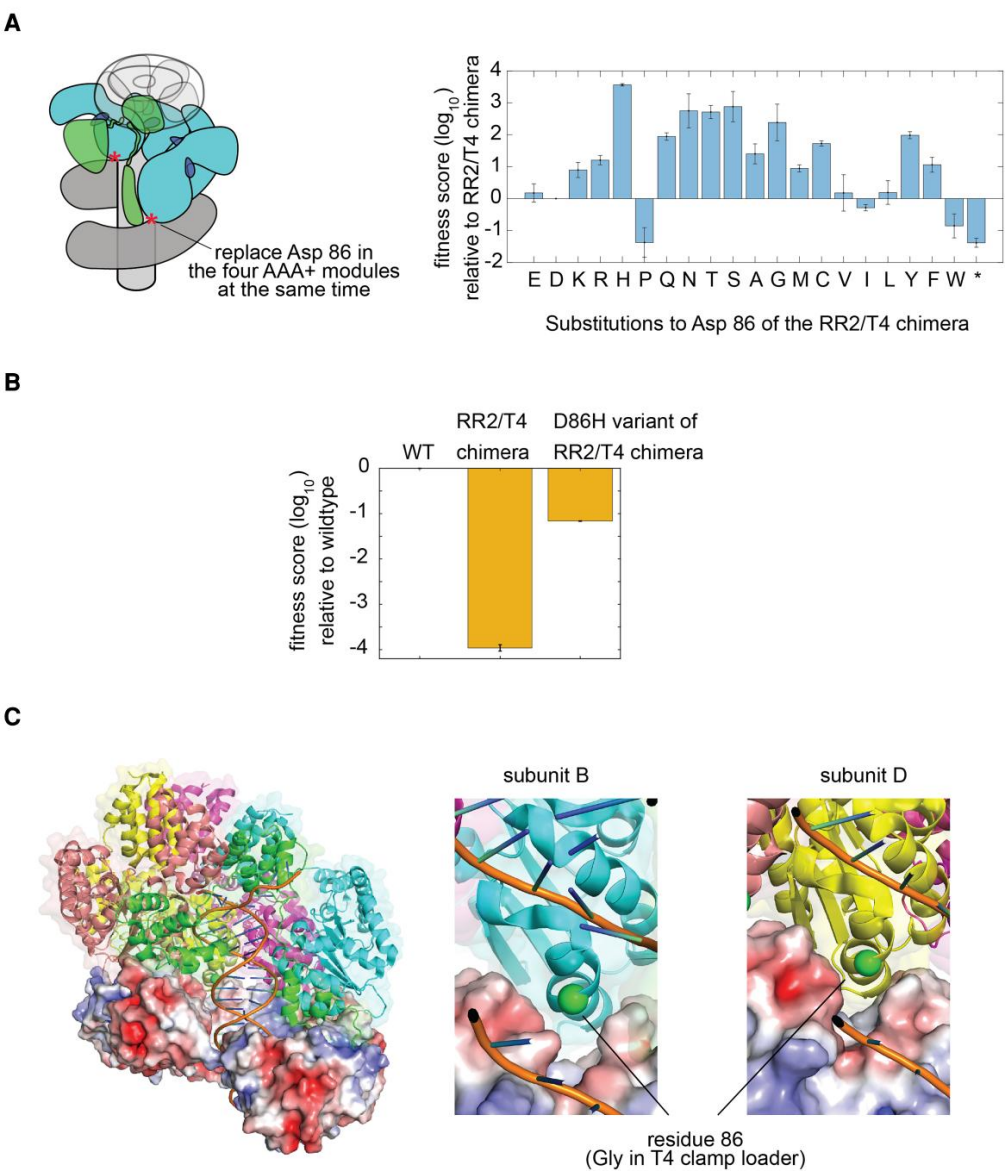
The fitness effect of mutating Asp 86 of the AAA+ module in the RR2/T4 chimeric clamp loader. a) A deep mutational scan of position 86 of the AAA+ module in the RR2/T4 chimeric clamp loader. b) Fitness scores of the RR2/T4 chimeric clamp loader and its D86H variant, relative to the wild-type T4 clamp loader. c) The location of residue 86 of the AAA+ module in the structure of the clamp loader bound to the sliding clamp and DNA (PDB ID: 3U60). The surface rendering of the sliding clamp is colored by electrostatic potential, calculated by using the Adaptive Poisson–Boltzmann Solver (APBS) Jurrus et al. (2018) ranging from −5.0 kT (red) to +5.0 kT (blue). All molecular illustrations were generated using Pymol (Schrödinger and DeLano 2020).

The Sanger sequencing covers only the genetic locus of the clamp loader and the sliding clamp and therefore will miss gain-of-function mutations that occur elsewhere on the genome. Therefore, we verified that the substitutions at Asp 86 in the RR2/T4 chimeric clamp loader do indeed result in a gain of fitness on their own. We carried out saturation mutagenesis of position 86 of the RR2/T4 chimera and tested all variants using the phage-propagation assay (Fig. 3a). Substitutions of Asp 86 by glycine, alanine, and asparagine residues—which appeared in the in vitro evolution experiment—result in 10-fold to 300-fold increases in phage propagation with respect to the RR2/T4 chimera (Fig. 3a). Importantly, the saturation mutagenesis data show that several other substitutions of Asp 86 that remove the negatively charged sidechain of Asp 86, such as substitutions by arginine, histidine, serine, cysteine, methionine, tyrosine, and phenylalanine, also increase the fitness of the RR2/T4 chimera.

We note that the D86H mutation, identified by saturation mutagenesis as causing the largest increase in fitness for the chimera, was not observed in the 15 independent trials of directed evolution. We suspect that the failure of the directed-evolution experiment to identify the importance of the D86H mutation is a result of mutational bias in the error-prone polymerase employed.

The histidine substitution at position 86 results in the most substantial increase in the fitness of phage (by ∼3,000-fold; Fig. 3a). In order to compare the fitness of the D86H variant of the RR2/T4 chimera with that of the wild-type T4 clamp loader, we performed a phage-propagation assay competing 3 clamp-loader variants: the wild-type T4 clamp loader, the RR2/T4 chimera, and the D86H variant of the RR2/T4 chimera (Fig. 3b). The conditions of the experiment are such that there is only one wave of phage infection, with very little uninfected cells at the onset of the experiment (multiplicity of infection [MOI] is 3). In these conditions, the RR2/T4 chimera propagates at a ∼10,000-fold slower than the T4 clamp loader. The fitness score of the D86H variant of the RR2/T4 chimera is −1.2 relative to the wild-type T4 clamp loader, which corresponds to ∼20-fold slower propagation compared with the wild-type variant. Thus, a single-point mutation (D86H) has repaired much of the deficiency of the RR2/T4 chimeric clamp loader.

The ability of several different substitutions of Asp 86 to rescue fitness suggests that the aspartate residue at this position in the RR2/T4 clamp loader may be involved in unfavorable interactions in the context of the chimeric clamp-loader complex and that the increased fitness is a consequence of removing these unfavorable interactions. Residue 86 is a part of helix α4, which, along with helices α5 and α6, constitutes the “central coupler” in AAA+ assemblies (Subramanian et al. 2021). The central coupler is a structurally contiguous unit that spans the AAA+ modules within the assembly, coupling the ATP-binding sites to DNA, to adjacent subunits, and to the sliding clamp. Helix α4 contacts the surface of the sliding clamp.

Inspecting the structure of the DNA-bound form of the T4 clamp loader (Kelch et al. 2011; Fig. 3c) provides a possible explanation for why the replacement of Asp 86 in the RR2/T4 chimera results in an increased fitness. For 2 of the 4 AAA+ modules in the structure, at positions B and D of the clamp-loader complex (Fig. 3c), residue 86—glycine in the T4 clamp loader—is positioned just above a region of negative electrostatic potential on the surface of the T4 sliding clamp. This suggests that replacing the T4 AAA+ module with that of the RR2 phage, which has the negatively charged sidechain of Asp 86 located at the interface with the clamp, would introduce an unfavorable electrostatic interaction and a decrease in the fitness of the clamp loader.

That Asp 86 causes unfavorable electrostatic interactions between the RR2/T4 chimeric clamp loader and the T4 sliding clamp is consistent with mutagenesis data for the T4 sliding clamp, as discussed below. The structures also suggest an additional explanation. Asp 86 is located next to Arg 85, a residue that is extremely sensitive to mutation in the phage-propagation assay and is highly conserved (Subramanian et al. 2021). The structure of the DNA-bound clamp-loader complex shows that Arg 85 contacts the phosphate backbone of DNA (Kelch et al. 2011). We modeled the structure of the AAA+ module of the RR2 clamp loader using AlphaFold (Jumper et al. 2021; Mirdita et al. 2022) and used it to carry out molecular dynamics simulations (6 independent trajectories, 2 μs each) of the free AAA+ module in the absence of DNA and the sliding clamp. When not able to interact with DNA, the simulations suggest that Arg 85 can make a stable ion pair with Asp 86 (supplementary fig. S5a and b, Supplementary Material online). It is possible that this interaction is also present in the context of the RR2/T4 chimera and the T4 sliding clamp, thereby reducing DNA affinity by competing with DNA for Arg 85, but this possibility awaits verification by additional experiments.

### Deep Mutational Scan of the Sliding Clamp

**Fig. 4.**
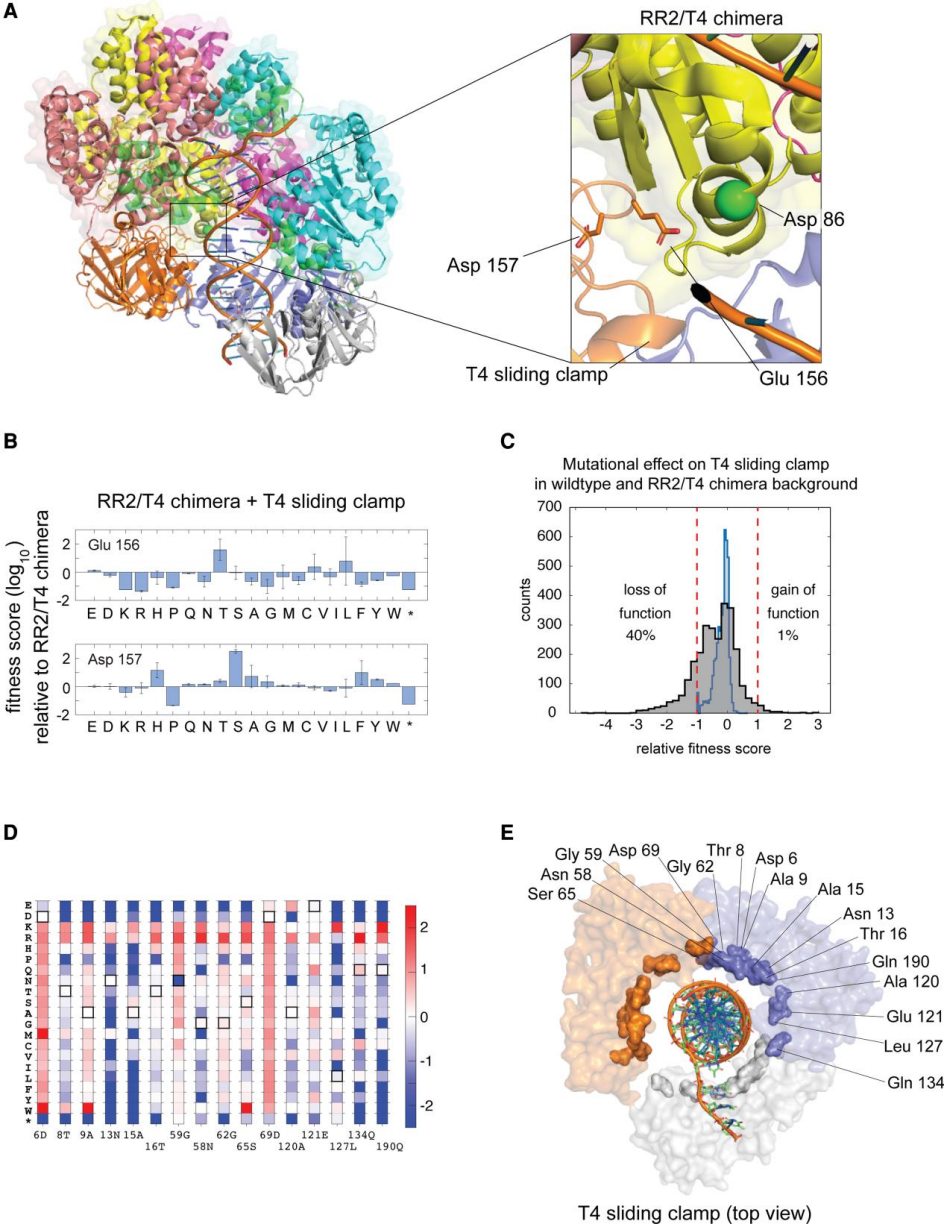
A deep mutational scan of the T4 sliding clamp in the context of the RR2/T4 chimeric clamp loader. a) Acidic residues of the T4 sliding clamp that could interact unfavorably with Asp 86 of the AAA+ module in the RR2/T4 chimeric clamp loader. b) Fitness scores for the mutational scan of the acidic residues in a). c) The distribution of mutational effects of the T4 sliding clamp in the context of the wild-type T4 clamp loader (blue) and the RR2/T4 chimeric clamp loader (black). d) The mutational profiles of residues in the T4 sliding clamp containing mutations that improve the fitness, by at least 10-fold, of the T4 phage propagating with the RR2/T4 chimeric clamp loader. e) Residues in d) were represented on the structure of the T4 sliding clamp (PDB ID: 3U60).

The idea that electrostatic repulsion between the clamp loader and the sliding clamp underlies the reduction in fitness of the RR2/T4 chimeric clamp loader is supported by the results of saturation mutagenesis of the T4 sliding clamp (supplementary fig. S6, Supplementary Material online). In this experiment, the T4 sliding clamp is subjected to saturation single-point mutagenesis, and phage propagation using the RR2/T4 chimeric clamp loader is measured. The fitness scores for the sliding clamp mutants are calculated relative to the phage propagating with the unmutated T4 sliding clamp and the RR2/T4 chimeric clamp loader. Residues Glu 156 and Asp 157 of the T4 sliding clamp contribute to the negative electrostatic potential on the surface of the clamp in the region adjacent to where the helix bearing Asp 86 interacts with the clamp (Fig. 4a). We observe that mutating these 2 residues increases fitness (Fig. 4b), with the E156T and D157S variants resulting in fitness scores of 1.6 ± 0.6 (∼10-fold faster phage propagation than that for the RR2/T4 chimera) and 2.5 ± 0.1 (∼300-fold faster phage propagation than that for the RR2/T4 chimera), respectively.

The data show that ∼1% (43 of 4,313) of the mutations to the T4 sliding clamp increase the rate of phage propagation by more than 10-fold relative to that with the RR2/T4 clamp loader (Fig. 4c). These gain-of-function mutations occur at 16 positions in the clamp, the mutational profiles of which are represented as a heatmap in Fig. 4d. The heatmap reveals a striking pattern: substitutions at these positions to arginine or lysine, residues that have positively charged sidechains, increase the fitness of the phage. In fact, any substitution that removes the negatively charged sidechain of Asp 6 and Asp 69 causes a fitness increase.

An explanation for this pattern emerges upon inspecting where these residues are located in the structure of the clamp loader in a complex with DNA and the sliding camp (Kelch et al. 2011; Fig. 4e). The 16 residues for which arginine and/or lysine substitutions increase fitness are proximal to DNA and are located on the inner surface of the T4 sliding clamp. Introducing positively charged residues at these positions can result in a favorable interaction with DNA. A multiple sequence alignment of ∼1,000 diverse sequences of the sliding clamp (supplementary fig. S7, Supplementary Material online) showed that arginine and lysine residues are sparsely sampled at these 16 positions. This suggests that the ability of the T4 clamp to increase the fitness of the phage through DNA interactions mediated by these regions represents an evolutionarily underutilized route to increase fitness, at least among extant sliding clamps.

### Deep Mutational Scan of the Clasp Subunit in the RR2/T4 Chimeric Clamp Loader

From a structural perspective, the clasp (A) subunit plays a crucial role in clamp-loader function because it bridges ATPase subunits B and E and plays a key role in recognizing the primer–template junction (Fig. 1a; Kelch et al. 2011). The clasp straddles the 2 domains of the sliding clamp that move apart when the clamp opens. Given its central role in the structure, it is surprising that the clasp subunit is highly tolerant to mutations in the context of the wild-type T4 system (Subramanian et al. 2021). This highlights the fact that when the clamp loader is operating under optimal conditions, it is most sensitive to mutations that break the integrity of the essential catalytic machinery.

In the RR2/T4 chimeric clamp loader, the clasp subunit is that of the T4 system. We reasoned that the reduced fitness of the RR2/T4 chimera would sensitize the clasp to mutations. We performed a deep mutational scan of the clasp subunit in the context of the RR2/T4 ch

imera and indeed observed a pattern of mutational sensitivity that is consistent with the expected structural importance of the clasp subunit (supplementary fig. S8, Supplementary Material online).

**Fig. 5.**
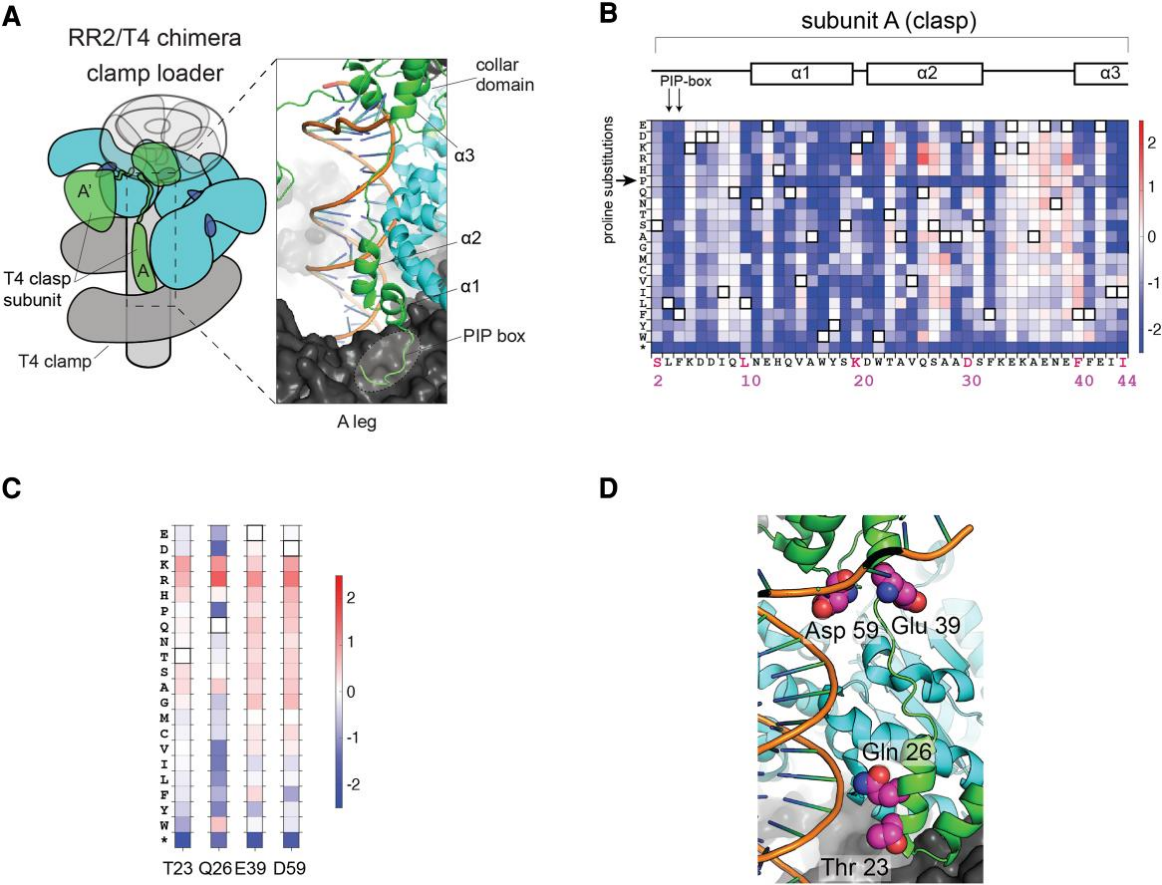
A deep mutational scan of the T4 clasp subunit in the context of the RR2/T4 chimeric clamp loader. a) The N-terminal arm of the T4 clasp subunit contacts the sliding clamp, DNA, and the B-subunit ATPase of the clamp loader. b) A deep mutational scan of the N-terminal arm of the T4 clasp subunit. c) The mutational profiles of residues in the T4 clasp subunit containing mutations that improve the fitness, by at least 10-fold, of T4 phage propagating with the RR2/T4 chimeric clamp loader. d) Residues in c) were represented on the structure (PDB ID: 3U60).

We highlight the mutational sensitivity of the clasp using a representative region of the clasp subunit, the N-terminal arm (residues 2 to 44, Fig. 5a). This segment of the clasp subunit contacts the sliding clamp near the location of the opening in the sliding clamp. It also interacts with the B-subunit of the clamp-loader complex and is situated adjacent to DNA. The importance of the structural integrity of the clasp subunit is highlighted by the fact that proline substitutions in helical regions, but not in loops, reduce the fitness of the RR2/T4 chimera (Fig. 5b and supplementary fig. S8, Supplementary Material online). Proteins that interact with the sliding clamp, including clamp loaders and DNA polymerases, engage the clamp using a conserved binding motif, the PCNA (Proliferating Cell Nuclear Antigen) interacting protein box, or the PIP box (Gulbis et al. 1996; Warbrick 1998). In the T4 phage, the principal interaction with the clamp utilizes a PIP box in the clasp subunit (Leu 3 and Phe 4; Fig. 5a). The data show that mutations to Leu 3 and Phe 4 substantially reduce (by more than 10-fold) the fitness of the RR2/T4 chimera (Fig. 5b).

Saturation mutagenesis of the clasp subunit identifies 8 gain-of-function substitutions that result in a >10-fold increase in phage propagation in the context of the RR2/T4 chimeric clamp loader. These substitutions occur in 5 residues in the clasp (Thr 23, Gln 26, Glu 39, Asp 59, and Asn 140; Fig. 5c). Except for Asn 140, the other 4 residues are proximal to DNA in the ternary structure of the clamp loader bound to DNA and the sliding clamp (Fig. 5d). The mutational profile at these 4 positions in the RR2/T4 chimera shows that introducing arginine or lysine, which can increase DNA affinity by interactions with the phosphate backbone, increases the rate of phage propagation (Fig. 5c). Thus, both the clasp subunit and the sliding clamp possess the ability to acquire single mutations that can increase DNA affinity. The fifth residue that shows gain-of-function in the clasp—Asn 140—is a noninterfacial residue, and the mechanism underlying the 20-fold increase in phage propagation caused by the N140T mutation is unclear at present.

### Deep Mutational Scan of the AAA+ Module of the RR2/T4 Chimeric Clamp Loader

We performed a deep mutational scan of the AAA+ module in the RR2/T4 chimeric clamp loader to identify mutations to the RR2 AAA+ module, which are beneficial in the chimeric context (supplementary fig. S9, Supplementary Material online). The data show that 9 positions (Pro 50, Asp 82, Asp 86, Cys 98, Val 106, Ala 112, Gly 113, Ala 115, and Gly 223) have at least 2 mutations that can cause a >10-fold increase in fitness. These 9 residues can be grouped into 3 sets (supplementary fig. S10, Supplementary Material online). The first set of residues (Asp 86, discussed earlier, and Cys 98) is on helix α4 and interacts with the sliding clamp, and substitutions to this set of residues are likely to improve fitness by increasing the affinity of the chimeric clamp loader for the clamp. The second set of residues (Pro 50 and Gly 223) belongs to a group of residues reported in another study of the T4 clamp loader (Marcus et al. 2024) that destabilize the DNA-free inactive structure, facilitating the transition to the DNA-bound active state. The third set of residues (Asp 82, Val 106, Ala 112, Gly 113, and Ala 115) contacts DNA-sensing residues (Lys 80 and Arg 111). Substitutions to this set of residues likely cause a repositioning of the DNA-sensing residues that increases DNA affinity and thereby the fitness of the phage.

### Conditional Neutrality in the RR2/T4 Chimeric Clamp Loader

**Fig. 6.**
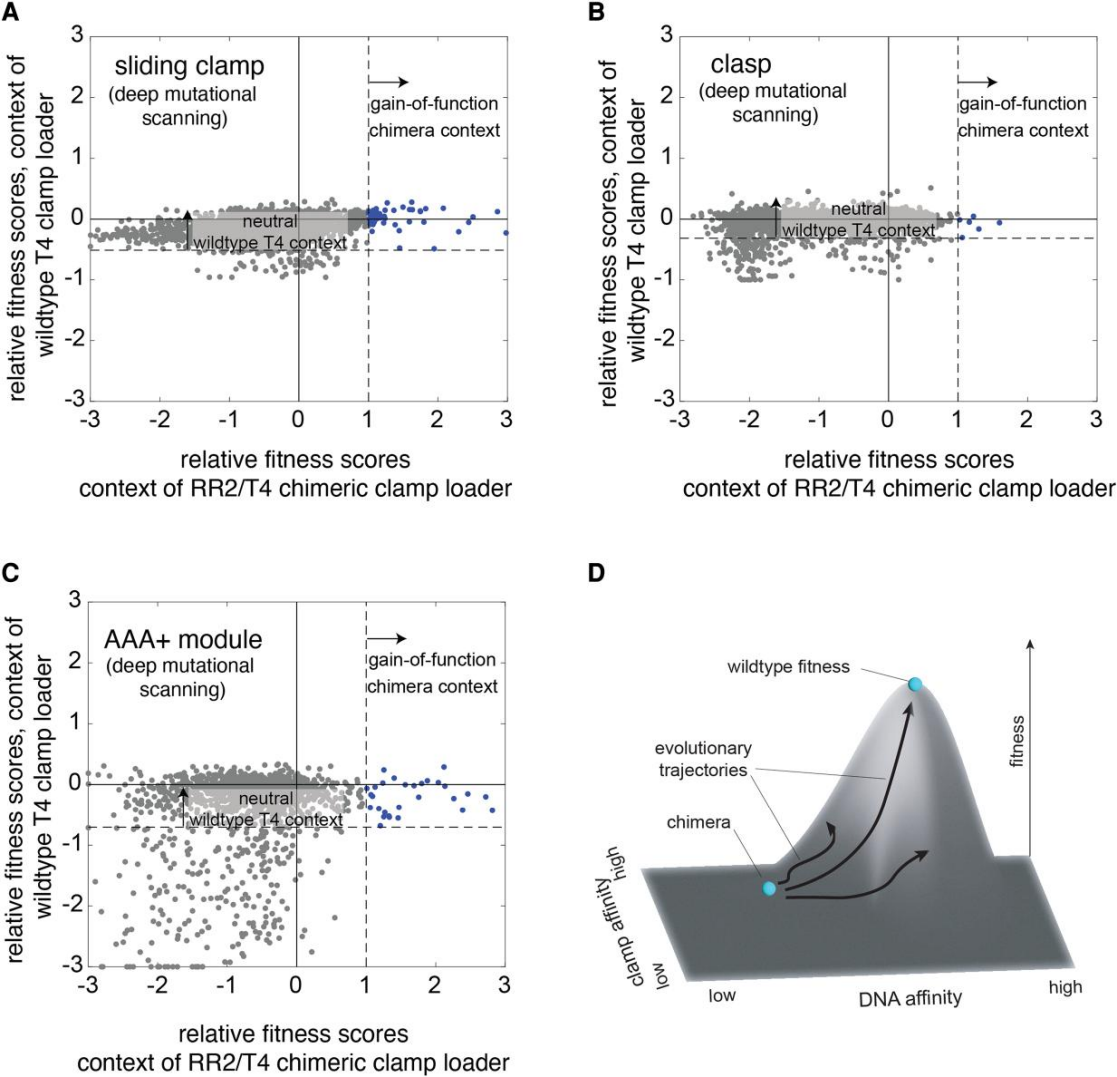
Conditionally neutral mutations in the clamp-loader complex. Scatter plots comparing the fitness effect of mutations in the context of the RR2/T4 chimeric clamp loader and the wild-type T4 clamp loader, for the sliding clamp a), the clasp subunit b), and the AAA+ module c). d) Schema depicting the idea that clamp-loader function can be optimized along many properties (DNA binding and clamp binding shown), but evolution balances these properties for optimal fitness for a given condition, say for T4 bacteriophage propagating in *E. coli*.

Conditional neutrality is a major emerging theme in protein adaptation that refers to mutations that are neutral in one setting but confer a fitness benefit on a new setting (Wagner 2008; Draghi and Plotkin 2011; Hayden et al. 2011; Zheng et al. 2019). The deep mutational scan of the T4 clamp-loader complex revealed many fitness-neutral mutations (Subramanian et al. 2021). We analyzed the results from deep mutational scanning of the RR2/T4 chimeric clamp loader to identify conditionally neutral mutations: mutations that are beneficial in the chimeric context and neutral in the context of the wild-type T4 clamp loader (Fig. 6). Mutations were deemed neutral in the wild-type T4 clamp-loader setting if their effect on fitness was within 3 SD from the mean of the fitness distribution of the synonymous variants of the wild-type T4 sequence. Using this threshold, the neutral range of fitness was within 4-fold of the wild type for the sliding clamp (supplementary fig. S11a, Supplementary Material online), within 3-fold of the wild type for the clasp (supplementary fig. S11b, Supplementary Material online), and within 5-fold of the wild type for the AAA+ module (supplementary fig. S11c, Supplementary Material online). Mutations in the RR2/T4 chimeric clamp-loader setting were considered beneficial if they caused at least a 10-fold increase in fitness relative to the fitness of the RR2/T4 chimera. We considered all positions in the AAA+ module in this analysis, irrespective of whether the positions are identical or different between the wild-type T4 and chimeric clamp loaders. Using these thresholds, all the beneficial mutations—53 mutations in the T4 sliding clamp (Fig. 6a), 6 mutations in the T4 clasp (Fig. 6b), and 33 mutations to the AAA+ module (Fig. 6c)—are conditionally neutral.

## Concluding Remarks

In this work, we studied the adaptive capacity of bacteriophage DNA polymerase clamp loaders by using a defective variant of the T4 clamp loader in which the AAA+ modules are replaced by those from a clamp loader in the RR2 phage. The resultant chimeric clamp loader causes the T4 phage propagation rate to drop by ∼5,000-fold. We subjected the T4-RR2 chimeric phage to directed evolution and found that point mutations to a single residue—Asp 86—in the RR2 AAA+ module can dramatically increase phage fitness. Based on the structure of the T4 clamp-loader complex, it appears that the mutation of Asp 86 repairs the chimeric clamp loader by reducing electrostatic repulsion with the T4 sliding clamp. A deep mutational scan of the T4 sliding clamp, the T4 clasp subunit, and the swapped-in RR2 AAA+ module, carried out in the context of the chimeric clamp loader, revealed that substitutions to residues proximal to DNA increase the fitness of the phage replicating with the chimeric clamp loader, presumably by tuning DNA interaction. Taken together, these results show how natural selection can exploit the adaptive potential latent in the clamp-loader subunits to improve fitness through single amino acid changes.

In a parallel study (Marcus et al. 2024), we investigated the adaptive capacity of the clamp loader by starting from a T4 clamp loader with a single mutation (D110C) in the conserved DEAD box motif that resulted in a mild (∼6-fold) reduction in function. We mapped the mutational response of the AAA+ module of the mutant clamp loader and found that this mild defect is rescued by a diverse set of mutations that are distributed throughout the AAA+ module. The locations of the mutations map to regions of conformational change between the active DNA-bound state of the clamp loader and the newly identified DNA-free inactive states, as shown by cryo-electron microscopy analysis (Marcus et al. 2024). These mutations, which individually have only a mild effect on fitness, were identified only because the fitness deficiency due to D110C is itself very mild. In contrast, the much more substantial defect in the RR2/T4 chimeric clamp loader is due to suboptimal interactions between noncognate components. Thus, the 2 studies highlight complementary mechanisms by which the clamp loader can alter its function—by modulating the strength of interaction between its components and its substrates (the sliding clamp and DNA) and by altering the free-energy balance between the active and the inactive conformations of the complex.

Conditionally neutral mutations play a pivotal role in facilitating the evolutionary diversification of proteins since they can preexist as neutral, standing genetic variation, waiting to reveal their beneficial effects when the environmental context changes (Luria and Delbrück 1943; Wagner 2005; Draghi and Plotkin 2011; Hayden et al. 2011; Dellus-Gur et al. 2013; Payne and Wagner 2014; Raman et al. 2016; Jayaraman et al. 2022). For example, the conditionally neutral mutations in the T4 sliding clamp can persist in a population of the T4 phage without affecting reproductive fitness, only to prove advantageous when chimeric clamp loaders arise due to a genetic transfer between 2 phage genomes. We revealed conditional neutrality in the clamp loader by using an artificially generated chimeric variant that is defective by construction. An intriguing avenue for future work is to study the generality of the conditionally neutral mutations: are the mutations that are beneficial in the chimera also advantageous in a differential context? The fact that mutations at residue Pro 50 of the AAA+ module of the T4 clamp loader are beneficial in the chimeric clamp loader as well as in the genetic background of the D110C mutation (Marcus et al. 2024) suggests that this might indeed be true.

Biological systems have many properties that can be optimized to increase certain aspects of their function. For the clamp loaders, this could be DNA binding, ATP binding/hydrolysis, clamp binding, or clamp release (Fig. 6d). Under a given set of conditions, evolution balances all these properties to maximize the fitness of the organism. Optimizing along just one property would reduce fitness—for example, a clamp loader that bound DNA too tightly would not release the clamp on DNA. In this study, we tipped the clamp loader off balance by creating chimeric clamp-loader variants that resulted in suboptimal intersubunit interactions. Remarkably, natural selection can find a new balance between these properties rather quickly, with point mutations sufficing to gain appreciable improvement in fitness.

### Key Resource Table

**Table:** 

Reagent type or resource	Designation	Source/reference	Identifiers	Additional information
Strain, strain background (bacteriophage T4)	T4^del^	Previous work (Subramanian et al. 2021)	…	https://benchling.com/s/seq-Vg4DZh83BOrbx3RgpaaC
Recombinant DNA reagent	CRISPR plasmid	Previous work (Subramanian et al. 2021)	…	https://benchling.com/s/seq-abaKV7JgTgAghRyR9ZIY
Recombinant DNA reagent	Nursing plasmid	Previous work (Subramanian et al. 2021)	…	https://benchling.com/s/seq-Itnyo8myS7Yfl5bWzaVh?m=slm-jri8sc6YFUOBYUNQPxIR
Recombinant DNA reagent	Recombination plasmid	Previous work (Subramanian et al. 2021)	…	https://benchling.com/s/seq-KdFkydUFMBrlRkdpORA3
Recombinant DNA reagent	Pol plasmid	This work	…	https://benchling.com/s/seq-cWvQfWGe4zeXcGtD8VmY?m=slm-EsOG72kK83Kk7fKnqETO
Recombinant DNA reagent	Helper plasmid	This work	…	https://benchling.com/s/seq-Ck1qhV3kd4JgbW1t0JLC?m=slm-RizOuNW9b8d2LENYCaR8
Recombinant DNA reagent	Mutator plasmid	This work	…	https://benchling.com/s/seq-xSUV1wORGSnRcsIU9l5n?m=slm-WgdIS9FXktidkOIdpeGL
Recombinant DNA reagent	Donor plasmid	This work	…	https://benchling.com/s/seq-Nnv5I7GNqFc1IyK2rnjD?m=slm-fFNttOSczD5izdwUL8Fu
Recombinant DNA reagent	WT T4 clamp loader	This work	…	https://benchling.com/s/seq-d4Pv8XhanDFJHN8T0grZ?m=slm-reuogjOWUJ1W5CHsNWUD
Recombinant DNA reagent	AAA+ module of RB69 clamp loader insert	This work	…	https://benchling.com/s/seq-qnZUjufEQTOfsGD8jLOi?m=slm-zxUzH9BUDwTw4UUACN1k
Recombinant DNA reagent	AAA+ module of RR2 clamp loader	This work	…	https://benchling.com/s/seq-Mw6la76oSFv4v6aopRKR?m=slm-60lwW2jvsxNdvRgbsSfB
Recombinant DNA reagent	AAA+ module of yeast RFC-C	This work	…	https://benchling.com/s/seq-wJxnuXp6Te7QRrxWNPOo?m=slm-fDrrhzGwSJXkQAEXFTVk
Recombinant DNA reagent	AAA+ module of yeast RFC-D	This work	…	https://benchling.com/s/seq-Opk7dnIVNgYhvd8OtFB6?m=slm-NpSWTx3pyK7VE3EUFp9U
Recombinant DNA reagent	AAA+ module of yeast RFC-E	This work	…	https://benchling.com/s/seq-NrdyOGODxAhh0MZv5v0i?m=slm-XLLoczjmy1Eheg6DcP72
Recombinant DNA reagent	NULL	This work	…	https://benchling.com/s/seq-YfDyKgxY1zYY1ZRKcQKz?m=slm-Kdu9VGgEYwx10fqp8IM8
Recombinant DNA reagent	rec_loader	This work	…	https://benchling.com/s/seq-NoHOAVvaMjLdaXwuX4aq?m=slm-o8XogGK0dcjvxIEwtRnE
Strain, strain background (bacteriophage T4)	T4^pol_del^	This work	…	https://benchling.com/s/seq-jxXhygOE38YBamD39uWE?m=slm-YdslLeeGFikUC5Yl6sgR
Strain, strain background (bacteriophage T4)	T4^chimera^	This work	…	https://benchling.com/s/seq-J60fgDjAJ3sp8q4BVz1X?m=slm-BCqRYAyA4dy23alXnN3V
Recombinant DNA reagent	Twist oligo pools	This work	…	https://www.dropbox.com/sh/wsi8wdoop39kdfs/AAC9jF2wg1ABXvTxJdq_nN6Ma?dl=0
Commercial assay or kit	MiSeq 500 cycles kit	Illumina	MS-102-2003	…
Software, algorithm	Kallisto	Bray et al. (2016)	…	https://pachterlab.github.io/kallisto
Software, algorithm	FLASH	Magoč and Salzberg (2011)	…	https://github.com/ebiggers/flash

## Materials and Methods

### Construction of Chimeric Variants of the Clamp Loader for the Phage-propagation Assay

The phage-propagation assay used to measure the ability of variant clamp-loader variants to support phage replication depends on a recombination plasmid that carries the genes encoding the clamp-loader subunits (Subramanian et al. 2021). We had previously generated a recombination plasmid containing the genes for the T4 clamp-loader complex, including gene 44 encoding the ATPase subunit and gene 62 encoding the clasp subunit (Subramanian et al. 2021). To generate chimeric variants of the clamp loader, we replaced residues 2 to 232 of the ATPase subunit of the T4 clamp loader with the corresponding residues from other AAA+ modules. DNA encoding other AAA+ modules was amplified from the genomes of *Saccharomyces cerevisiae*, bacteriophage RB69, and bacteriophage RR2. We introduced the BamHI restriction site (GGATCC) in gene 44, between regions encoding the AAA+ module and the collar domain for ease of cloning. We generated a NULL variant of the clamp loader by introducing 3 stop codons after the start codon of gene 44. Hyperlinks to the annotated plasmid sequences are provided in the key resource table.

### Construction of Single-Mutant Libraries

We constructed comprehensive single-mutant libraries of the clamp, clasp, and AAA+ module of the chimeric clamp-loader complex using previously described methods (Subramanian et al. 2021). The primers used to generate the mutant library for the sliding clamp were reused to generate the library in the recombination plasmid containing the chimera. Mutant libraries for the clasp subunit and the AAA+ module were generated using oligonucleotide pools ordered from Twist Biosciences (San Francisco, CA, USA). The AAA+ module library was generated using 10 pools, each encoding 480 sequences corresponding to 24 positions. The mutant library for the clasp subunit was generated using 3 pools, each encoding ∼1,300 sequences corresponding to 65 positions. The sequences of the oligonucleotide pools are available here.

### Phage-propagation Assay

The phage-propagation assay was performed as described in previous work (Subramanian et al. 2021). Briefly, the plasmid library encoding the RR2/T4 chimeric clamp-loader variants was transformed into bacterial cells containing the CRISPR plasmid and recovered in an overnight growth in selective media. Approximately one hundred microliters of the dense, overnight culture was inoculated into fresh ∼110 ml Luria-Bertani broth (LB) broth to start an exponentially growing culture. Plasmid was extracted from ∼10 ml of the culture (OD_600_ = 0.1), and the DNA was set aside as the starting library. Phage propagation was started by adding ∼10^8^ T4^del^ phage particles to the remainder of the culture (∼100 ml). This corresponds to a MOI of 0.1. The phage infection was allowed to proceed for ∼24 h at 37 °C in a shaking incubator and then allowed to sit on the bench for the cell debris to settle. Approximately one milliliter of the culture was centrifuged to remove cell debris, and the supernatant was saved as the selected phage library. DNA samples were prepared for Illumina sequencing through 3 sequential polymerase chain reaction (PCR) steps, as described previously (Subramanian et al. 2021), and sequenced on the MiSeq sequencer using the 500 cycles kit.

### Analysis of the Raw Sequencing Data

The different clamp-loader sequence variants present in the sequencing data were quantified using Kallisto version 0.48.0 obtained from GitHub. The Kallisto index was built, using default parameters, from a custom fasta file containing all possible unique DNA sequences encoding the clamp-loader variants. Relative fitness scores were calculated using custom MATLAB scripts available here.

### Generation of the T4^chimera^ Strain for Directed Evolution Through Phage Propagation

The T4^chimera^ strain used in the directed-evolution trials was generated from the T4 bacteriophage in 2 steps. In the first step, we generated a new strain of T4, termed T4^pol_del^, in which the genes encoding the polymerase and the clamp-loader complex are deleted. In the second stage, the genes encoding the RR2/T4 chimeric clamp-loader complex were introduced into T4^pol_del^ to generate the T4^chimera^.

### Generation of T4^pol_del^

In our previous work, we developed a method to delete essential genes from the genome of bacteriophage T4. We had used the method to delete the genes encoding the clamp loader and the sliding clamp (Subramanian et al. 2021). In this study, we apply the same method to generate T4^pol_del^, the strain of T4 in which the genes for the DNA polymerase and the clamp-loader complex are deleted. The genes to be deleted are adjacent to each other on a ∼6-kb stretch of the T4 genome (32,886 to 30,341 bp, numbered according to the GenBank file with accession number AF158101). We used a CRISPR system to generate a cut in this span and also used a homologous recombination to replace the span with a short, 20-bp segment to generate T4^pol_del^. The newly inserted 20-bp segment can be targeted using CRISPR, enabling us to insert the chimera at this site in order to generate the T4^chimera^.

We infected *E. coli* BL21 cells carrying 3 plasmids with wild-type T4 bacteriophage particles to generate T4^pol_del^. The first plasmid, termed the CRISPR T4 plasmid, encodes CRISPR-Cas12a and a guide RNA targeting the region to be deleted. The second plasmid, termed the donor plasmid, contains a new CRISPR-Cas12a recognizable site (TTTACCGGGAGGAAGATATAGCAC, PAM sequence is underlined) that is flanked on both sides by ∼1-kb homologous arms. The homologous arms are identical to the regions on either side of the target locus on the T4 genome. The third plasmid, termed the helper plasmid, encodes a copy of the T4 genes that are to be deleted and can therefore support the propagation of T4^pol_del^. We isolated T4^pol_del^ plaques and verified that the isolated phage is unable to propagate in bacteria unless the cells carry the helper plasmid. A Sanger sequencing of the polymerase genetic locus in the isolated phage provided confirmatory evidence that we had indeed generated T4^pol_del^.

### Generation of the T4^chimera^

We constructed a new recombination plasmid, termed rec_loader plasmid, to introduce the chimera into T4^pol_del^. The rec_loader plasmid contains the gene encoding the T4 sliding clamp and the genes encoding the ATPase and clasp subunits of the chimera. These genes are flanked by arms of homology in order to recombine into the polymerase locus on the genome of T4^pol_del^ and generate the T4^chimera^.

We infected *E. coli* BL21 cells carrying 3 plasmids with T4^pol_del^ to generate the T4^chimera^. The first plasmid is the CRISPR plasmid that targets the newly inserted segment on T4^pol_del^. The second plasmid is the rec_loader plasmid, discussed above, that encodes the genes for the RR2/T4 chimeric clamp-loader complex. The third plasmid is the pol plasmid, a plasmid encoding the T4 DNA polymerase that is required to propagate the T4^chimera^. We isolated phage particles from the infection and used Sanger sequencing to verify the correct genomic integration of the genes corresponding to the chimera.

### Directed Evolution Through Phage Propagation

Directed evolution was performed by passaging phage populations in bacteria carrying a plasmid-borne copy of the D219A variant of the T4 DNA polymerase, an error-prone variant having a ∼1,000-fold higher mutation rate than the wild-type polymerase (Frey et al. 1993). Each trial of directed evolution was started with ∼10^5^ particles of the T4^chimera^ added to 2 ml of *E. coli* BL21 cells with the mutator plasmid (key resource table) that are exponentially growing with OD_600_ between 0.1 and 0.2. The phage was allowed to propagate in this culture for 8 h overnight at 37 °C. The culture was centrifuged to precipitate cell debris, and 1 ml of the supernatant was saved for analysis. Two microliters of the supernatant was used to inoculate a fresh, 2-ml culture of bacteria with the mutator plasmid.

To perform Sanger sequencing, 1 µl of the supernatant was used as a template in a 20-µl PCR mix to amplify the clamp-loader locus, using primers with sequences TAACCAAAATAGCGATTTTC and TTGCTGCTCAAATTGTTG.

### Molecular Dynamics Simulations

We modeled the AAA+ module of the RR2 clamp loader using AlphaFold (Jumper et al. 2021). Molecular dynamics simulations were set up similar to our previous work (Subramanian et al. 2021). Histidine protonation states were inferred using the H++ web server (Anandakrishnan et al. 2012). We solvated the model with TIP3P water within a truncated octahedron 15 Å larger than the AAA+ module and neutralized with Na+ using tleap (Case et al. 2019) and added 150 mM of NaCl based on this initial volume. Six independent trajectories with different random seeds were started with this model. In each case, 1 ns Langevin dynamics (1/ps friction coefficient) with a 1-fs time step was used to equilibrate the system in NVT (constant-temperature, constant-volume ensemble) at 310.15 K within pmemd on an NVIDIA A40 GPU. After this, a Monte Carlo barostat was used with a 2-fs time step to equilibrate the NPT (constant-temperature, constant-pressure ensemble) for 1 ns. Then, the models were subjected to equilibration for a total of 2 µs, and distances between the guanidino carbon of Arg 85 and the Cγ of Asp 86 were calculated for the equilibration trajectory.

## Supplementary Material

Supplementary material is available at *Molecular Biology and Evolution* online.

## Supplementary Material

msae013_Supplementary_Data

## Data Availability

The Illumina sequencing data generated in this study have been deposited in the Sequence Read repository with BioProject accession number PRJNA1076877. Scripts to analyze the data and the enrichment values calculated can be accessed from our Github repository: https://github.com/KuriyanLab/clamp_loader_adaptive_capacity.
