# Supplementary material for: Adaptive Capacity of a DNA Polymerase Clamp-loader ATPase Complex
Source: Mol Biol Evol. 2024 Jan 31;41(3):msae013. doi: 10.1093/molbev/msae013 (PMC10924251; doi:10.1093/molbev/msae013)
Supplement: msae013_Supplementary_Data [file msae013_supplementary_data.pdf]

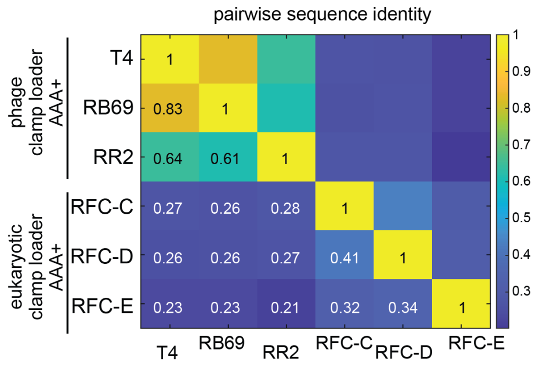

Supplementary Figure 1. Sequence similarity between AAA+ modules in the constructed chimeric clamp loaders. Pairwise sequence identitity between AAA+ modules used in the study.

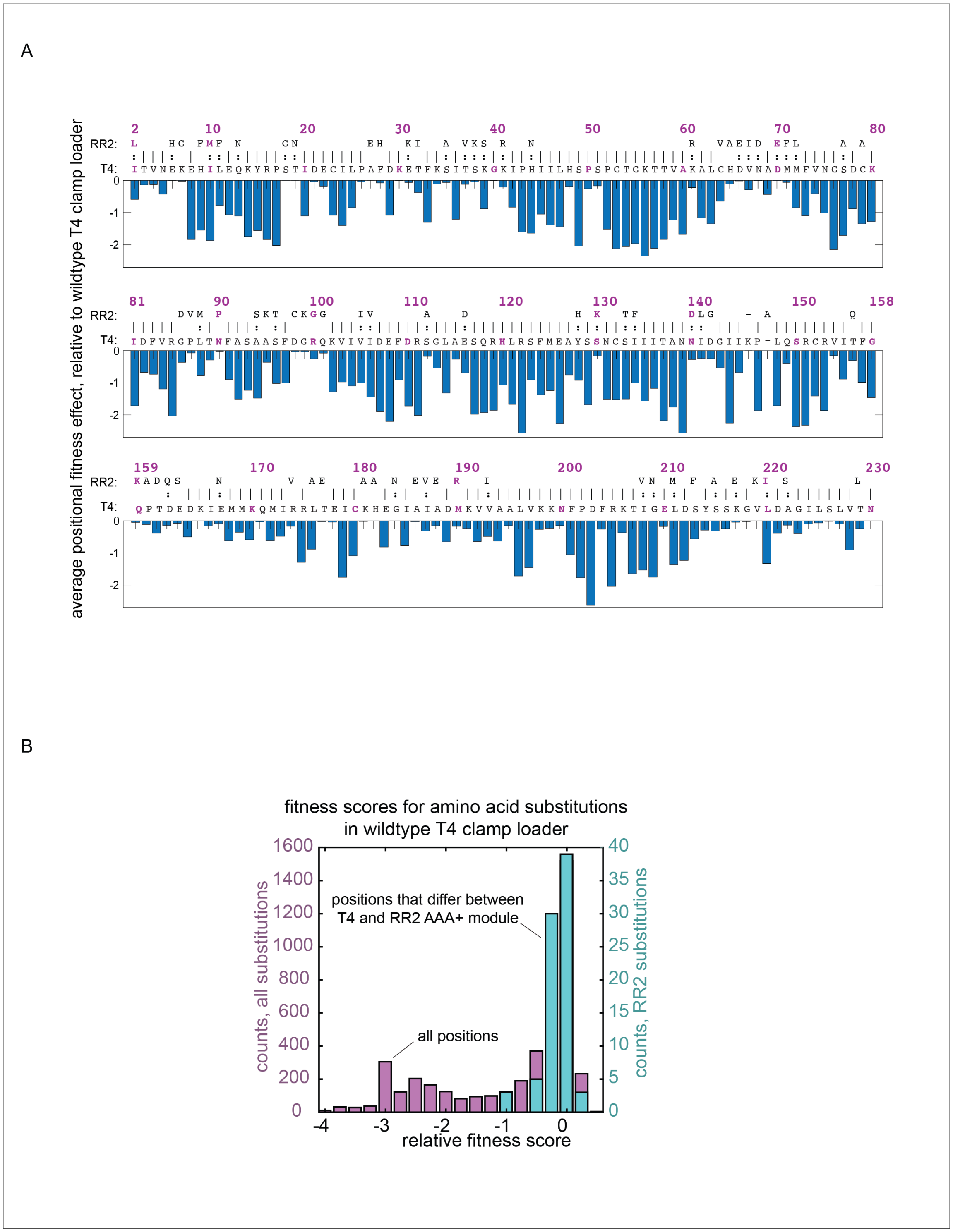

Supplementary Figure 2. Mutational effects in the AAA+ module of the wildtype T4 clamp loader. (A) Average effect mutating each position of the AAA+ module in the wildytpe T4 clamp loader, based on fitness values from (Subramanian et al. 2021)).The sequence of the RR2 AAA+ module is aligned with T4 AAA+ module. (B) Distribution of fitness effects, for the wildtype T4 clamp loader, from introducing individual substitions to each of the differing residues in the corresponding RR2 AAA+ module.

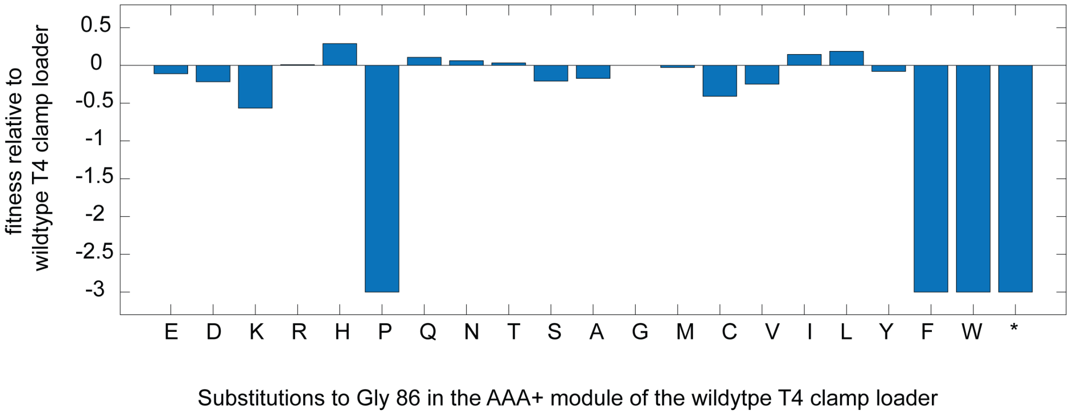

Supplementary Figure 3. Effect of single amino acid substitutions at position 86 of the ATPase subunit of the wildtype T4 clamp loader. Fitness values from (Subramanian et al. 2021)).

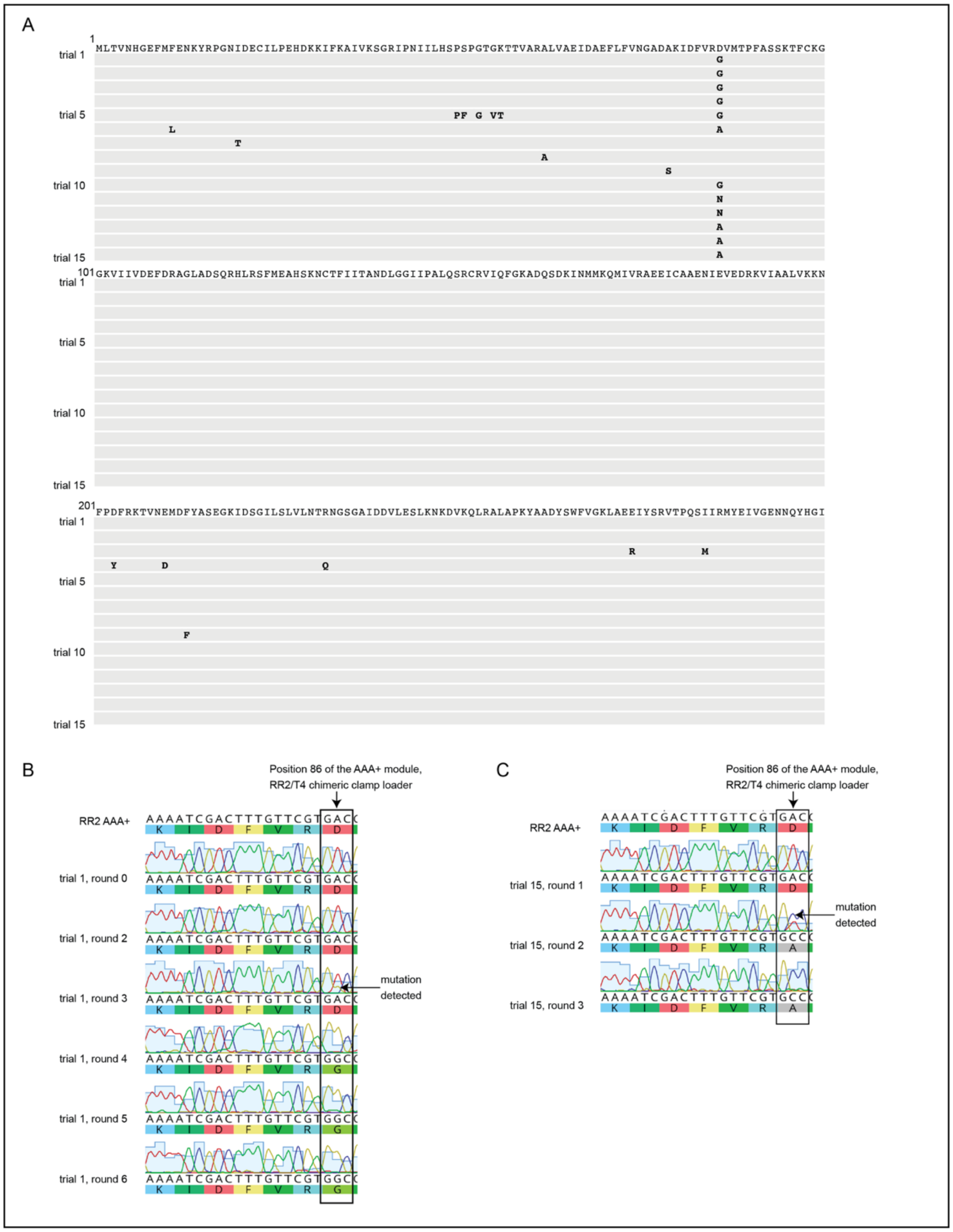

Supplementary Figure 4. Sanger Sequencing of *in vitro* evolution trials. (A) Sequencing of the AAA+ module of the RR2/T4 chimeric clamp loader locus in T4^{chimera}, after round 10 from each of the 15 parallel trials of *in vitro* evolution. Changes to the starting protein sequence is shown. (B) and (C) Sanger sequencing chromatograms for the early rounds of trial 1 and trial 15, for the 21-nucleotide segment ending at the codon corresponding to residue 86 of the AAA+ module of the RR2/T4 chimeric clamp loader locus.

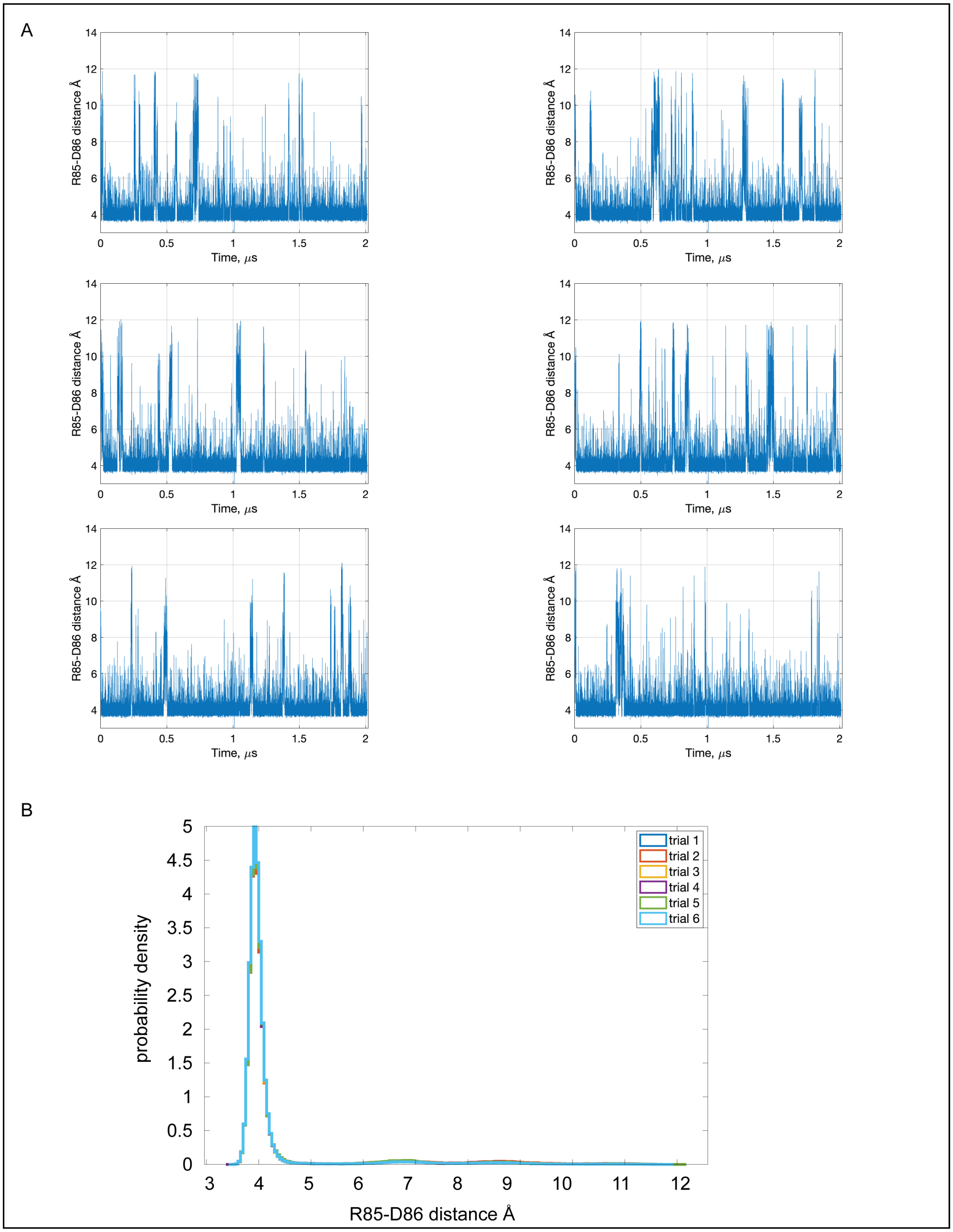

Supplementary Figure 5. Molecular dynamics simulations of the AAA+ module of the RR2 clamp loader. (A) The distance between the guanidino group of Arg 85 and the γ-carbon (sidechain carboxyl group) of Asp 86 are plotted for a 2μs simulation for six molecular dynamics trajectories. (B) Probability density of the distance between Arg 85 and Asp 86, from **A**.

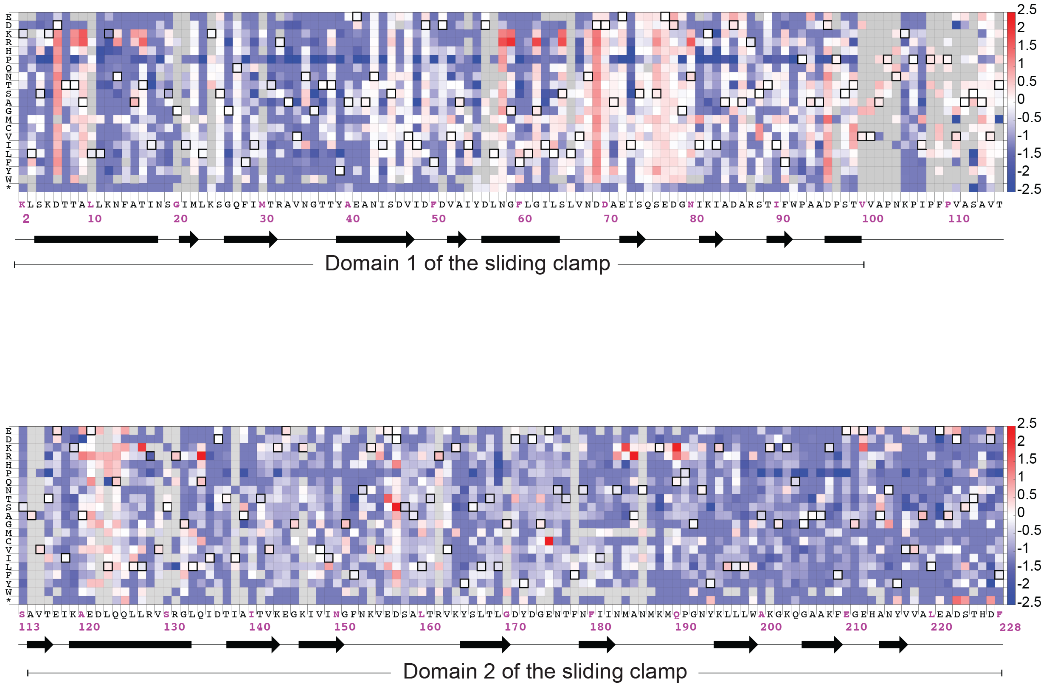

Supplementary Figure 6. Mutational sensitivity of the T4 sliding clamp in the context of the RR2/T4 chimeric clamp loader. Fitness scores are calculated relative to the unmutated T4 sliding clamp in the context of the RR2/T4 chimeric clamp loader. Amino acid substitutions that result in the wild-type sequence are denoted by pixels with a black border. Gray pixels denote mutations with insufficient counts (fewer than 10 counts) when sequencing the plasmid library. The secondary structure of the wild-type sequence is indicated below the wild-type sequence, with α helices as rectangles and β strands as arrows.

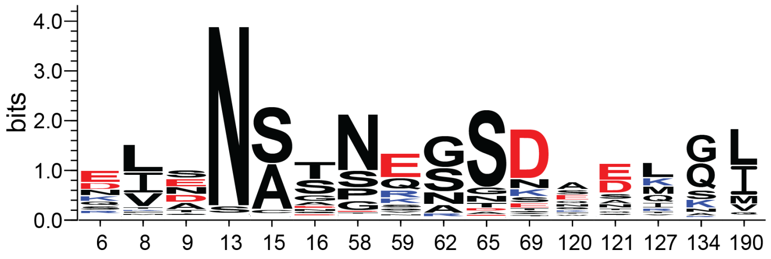

Supplementary Figure 7. Sequence logo of the 16 positions in the T4 sliding clamp in which substitutions can result in at least a 10-fold increase in phage propagation. Residues with acidic side chains (Asp and Glu) are shown in red, residues with basic sidechains (Arg and Lys) are shown in blue. Asn 13, a residue in domain 1 of the sliding clamp that forms a hydrogen-bonded interaction with the amide backbone of domain 2 at residue 204 in the T4 clamp loader, shows a sequence signature of strong evolutionary conservation.

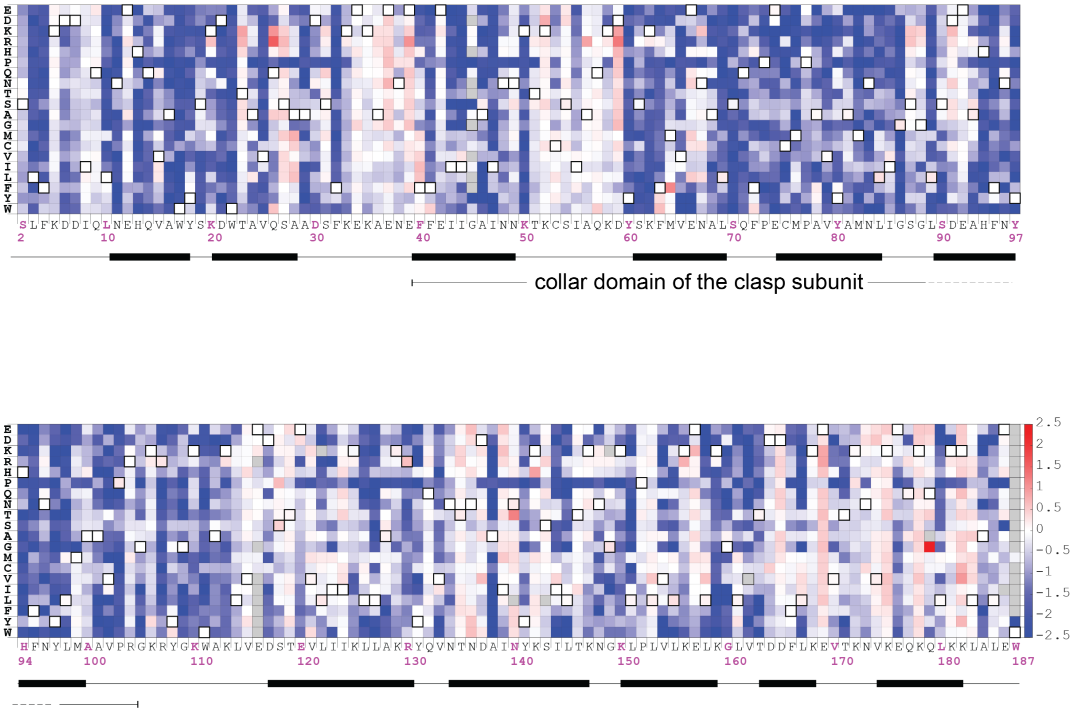

Supplementary Figure 8. Mutational sensitivity of the T4 clasp subunit in the context of the RR2/T4 chimeric clamp loader. Fitness scores are calculated relative to the unmutated RR2/T4 chimeric clamp loader. Amino acid substitutions that result in the wild-type sequence are denoted by pixels with a black border. The secondary structure of the wild-type sequence is indicated below the wild-type sequence, with α helices as rectangles and β strands as arrows.

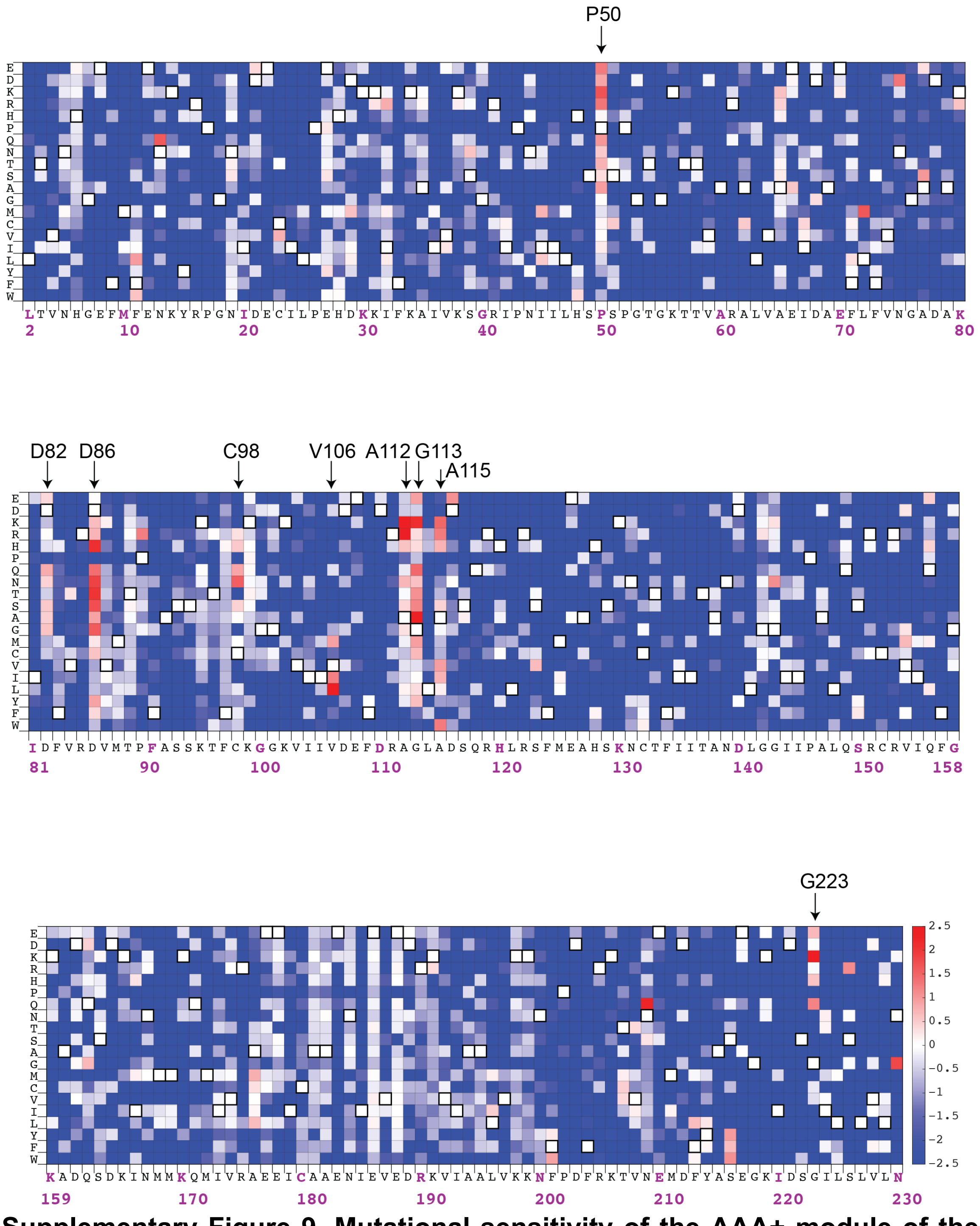

Supplementary Figure 9. Mutational sensitivity of the AAA+ module of the RR2/T4 chimeric clamp loader. Fitness scores are calculated relative to the unmutated RR2/T4 chimeric clamp loader. Amino acid substitutions that result in the wild-type sequence are denoted by pixels with a black border. The secondary structure of the wild-type sequence is indicated below the wild-type sequence, with α helices as rectangles and β strands as arrows.

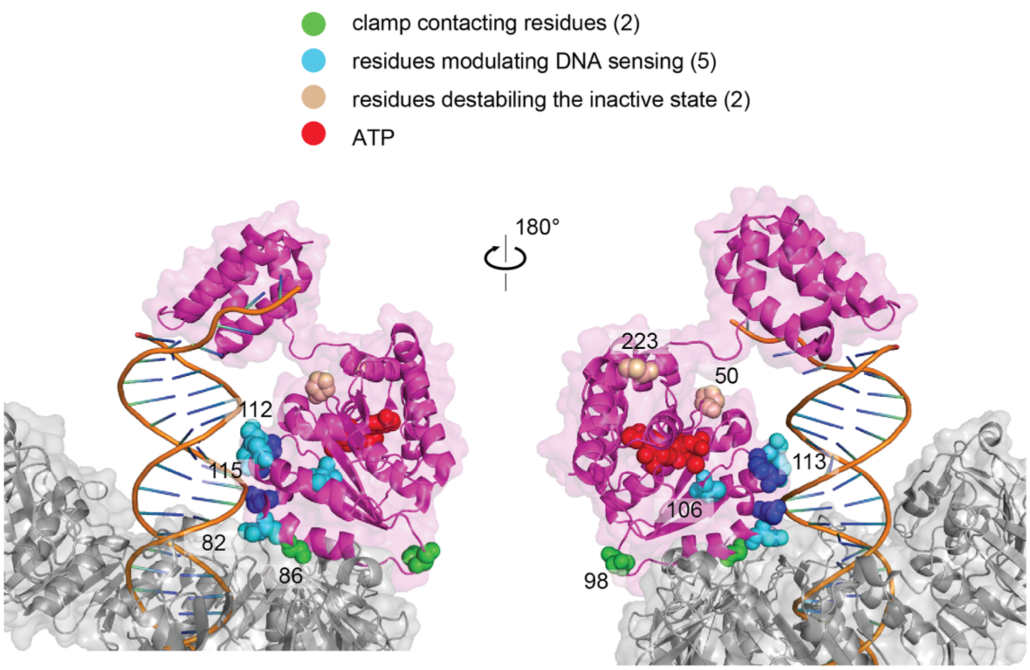

Supplementary Figure 10. Gain-of-function mutations in the AAA+ module of the RR2/T4 chimeric clamp loader. The spatial location of residues in the AAA+ module of the RR2/T4 clamp loader in which substitutions can result in at least 10-fold increase in phage propagation (PDB ID: 3U60). Residues are grouped into 3 set: clamp contacting residues, DNA contacting residues and residues that can destabilize the DNA-free inactive state of the clamp loader.

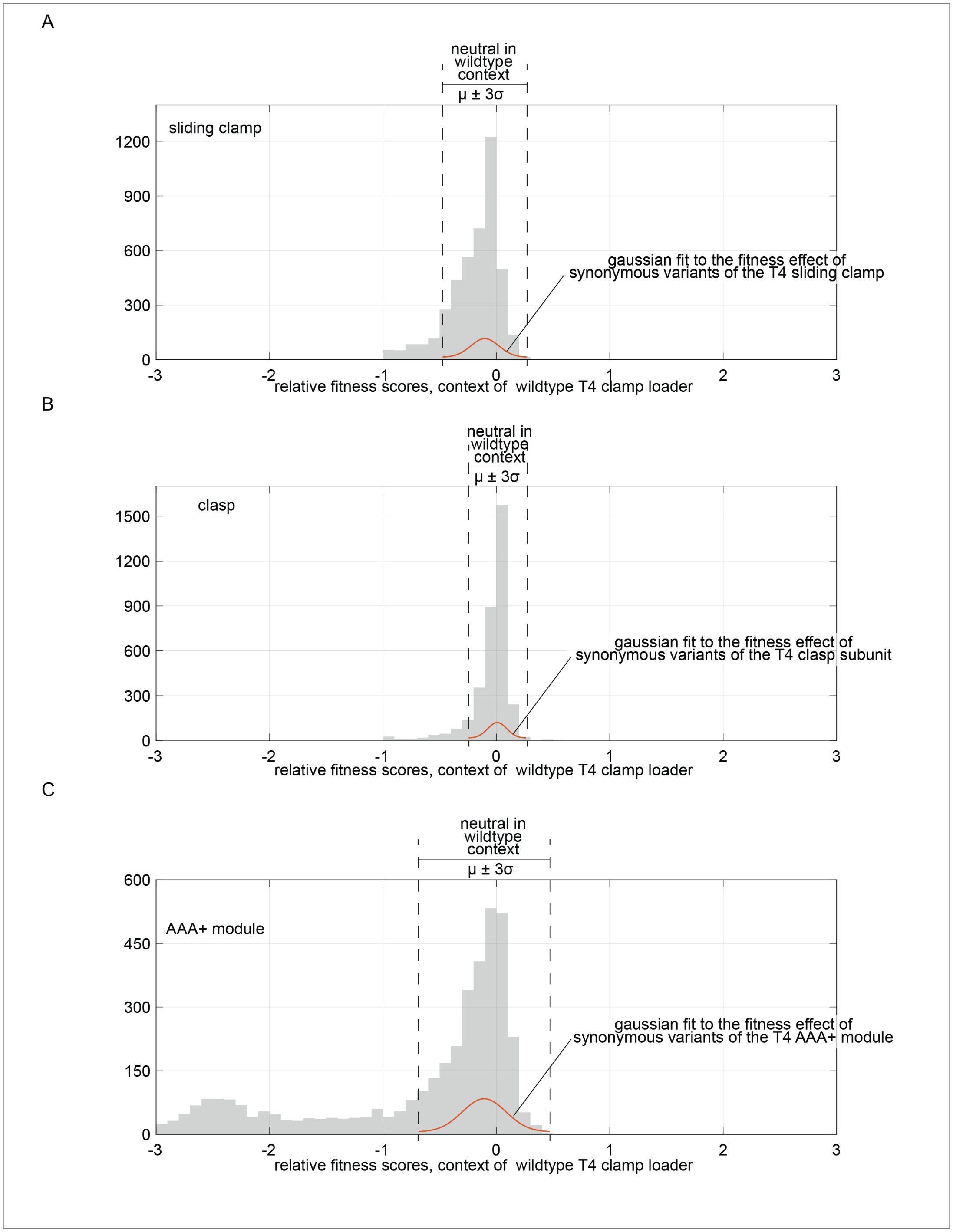

Supplementary Figure 11. Determining the threshold for neutral mutations in the wildytpe T4 clamp-loader complex. The distribution of fitness effects of point mutations to the sliding clamp (A), the clasp (B) and the AAA_ module (C) of the wildytpe T4 clamp-loader complex are plotted as histograms (gray). A gaussian fit to the fitness effect of synonymous variants of the reference sequence is shown in red, along with the three standard deviations threshold for the fit.
